# The Effects of GC-Biased Gene Conversion on Patterns of Genetic Diversity among and across Butterfly Genomes

**DOI:** 10.1093/gbe/evab064

**Published:** 2021-03-24

**Authors:** Jesper Boman, Carina F Mugal, Niclas Backström

**Affiliations:** Evolutionary Biology Program, Department of Ecology and Genetics (IEG), Uppsala University, Sweden

**Keywords:** genetic diversity, GC-biased gene conversion, Lepidoptera, linked selection, mutation bias

## Abstract

Recombination reshuffles the alleles of a population through crossover and gene conversion. These mechanisms have considerable consequences on the evolution and maintenance of genetic diversity. Crossover, for example, can increase genetic diversity by breaking the linkage between selected and nearby neutral variants. Bias in favor of G or C alleles during gene conversion may instead promote the fixation of one allele over the other, thus decreasing diversity. Mutation bias from G or C to A and T opposes GC-biased gene conversion (gBGC). Less recognized is that these two processes may—when balanced—promote genetic diversity. Here, we investigate how gBGC and mutation bias shape genetic diversity patterns in wood white butterflies (*Leptidea* sp.). This constitutes the first in-depth investigation of gBGC in butterflies. Using 60 resequenced genomes from six populations of three species, we find substantial variation in the strength of gBGC across lineages. When modeling the balance of gBGC and mutation bias and comparing analytical results with empirical data, we reject gBGC as the main determinant of genetic diversity in these butterfly species. As alternatives, we consider linked selection and GC content. We find evidence that high values of both reduce diversity. We also show that the joint effects of gBGC and mutation bias can give rise to a diversity pattern which resembles the signature of linked selection. Consequently, gBGC should be considered when interpreting the effects of linked selection on levels of genetic diversity.

**Significance**

Recombination is a process which occurs during the formation of egg and sperm and leads to the assembly of novel chromosomes from maternal and paternal copies. This has widespread implications for the evolution of genetic variants by breaking the physical linkage between selected and neutral sites on the same chromosome. Here, we show that a balance between the neutral evolutionary processes of gene conversion and variable mutation rate can result in a similar pattern as widespread selection on linked sites. Consequently, this study adds to our understanding of the complex interaction between selective and neutral forces governing genetic diversity patterns.

## Introduction

The neutral theory of molecular evolution postulates that the majority of genetic differences within and between species are due to selectively neutral variants (Kimura 1983; Jensen et al. 2019). Consequently, the level of genetic variation within populations (θ) is expected to predominantly be determined by the effective population size (*N*_e_) and the mutation rate (µ) according to the following relationship: θ* *=* *4 *N*_e_µ. Indeed, differences in life-history characteristics (as a proxy for *N*_e_) have been invoked as explanations for the interspecific variation in genetic diversity among animals (Romiguier et al. 2014). In addition, among butterflies, body size is negatively associated with genetic diversity (Mackintosh et al. 2019). Lewontin (1974) noted that the range of observed values of *N*_e_ estimated from genetic diversity measures is smaller than the range of census population sizes, *N*_c_ (Lewontin’s paradox; Lewontin 1974; Kimura 1983; Nevo et al. 1984; Frankham 1995). Lower *N*_e_ compared with *N*_c_ may be caused by more efficient selection and subsequently reduced genetic diversity in large compared with small populations (Corbett-Detig et al. 2015). In particular, selection affects the allele frequency of linked neutral sites (commonly referred to as linked selection or genetic draft) and reduces their diversity (Maynard Smith and Haigh 1974; Charlesworth et al. 1993).

However, linked selection in itself is not necessarily the solution to Lewontin’s paradox. It has been noted that *N*_e_* *=* N*_c_ is true only for a population in mutation–drift equilibrium (Galtier and Rousselle 2020). Furthermore, changes in population size may amplify the effects of linked selection and the relative importance of selection and demography is an ongoing debate (Corbett-Detig et al. 2015; Coop 2016; Kern and Hahn 2018; Jensen et al. 2019). This debate concerns the fate and forces affecting an allele while segregating in a population. Although this is important for resolving Lewontin’s paradox, it only addresses variation in *N*_e_, which is but a part of the puzzle of genetic diversity. As noted above, variation in the occurrence of mutations also influences genetic diversity. The general pattern observed is a negative relationship between mutation rate and *N*_e_ among species (Lynch et al. 2016)*.* This may be explained by a selective pressure for reducing the overall mutation rate resulting from the distribution of fitness effects of new mutations being dominated by deleterious mutations (Eyre-Walker and Keightley 2007; Lynch et al. 2016). However, mutation rates vary only over roughly one order of magnitude in multicellular eukaryotes (Lynch et al. 2016) and appear less important than *N*_e_ for interspecific differences in genetic diversity.

Genetic diversity can also vary among genomic regions. The determinants of such regional variation are currently debated, but variation in mutation rate (Hodgkinson and Eyre-Walker 2011; Smith et al. 2018) and linked selection have both been considered (Cutter and Payseur 2013; Corbett-Detig et al. 2015). Higher rates of recombination are expected to reduce the decline in diversity experienced by sites in the vicinity of a selected locus. Begun and Aquadro (1992) showed for example that genetic diversity was positively correlated with the rate of recombination in *Drosophila melanogaster.* Their finding validated the impact of selection on linked sites, previously predicted by theoretical work (reviewed in Comeron 2017). Since then, multiple studies have found a positive association between recombination rate and genetic diversity (Begun and Aquadro 1992; Nachman 1997; Kraft et al. 1998; Cutter and Payseur 2003; Lohmueller et al. 2011; Langley et al. 2012; Cutter and Payseur 2013; Mugal et al. 2013; Burri et al. 2015; Corbett-Detig et al. 2015; Wallberg et al. 2015; Martin et al. 2016; Pouyet et al. 2018; Talla, Soler, et al. 2019; Castellano et al. 2020). The positive correlation between diversity and recombination may, however, be caused by factors other than selection on linked sites. Recombination may for instance be mediated towards regions of higher genetic diversity (Cutter and Payseur 2013), or have a direct mutagenic effect (Hellmann et al. 2005; Arbeithuber et al. 2015; Halldorsson et al. 2019). Additionally, analytical evidence suggests that the interplay between mutation bias and a recombination-associated process, GC-biased gene conversion (gBGC), can increase nucleotide diversity (McVean and Charlesworth 1999). GC-biased gene conversion in itself will—like directional selection—reduce diversity of segregating variants. If we additionally consider the long-term effect of gBGC and the concomitant increase in GC content, then genetic diversity may rise as a consequence of gBGC through increased mutational opportunity in the presence of an opposing mutation bias (McVean and Charlesworth 1999; Vogl and Mikula 2021). To fully understand the effects of recombination on genetic diversity, we must therefore consider both gBGC and opposing mutation bias, in addition to the much more recognized influence of linked selection. In other words, what relationship do we expect between recombination and genetic diversity in the presence of nonadaptive forces such as gBGC and mutation bias?

To understand the mechanistic origins of gBGC, we must first consider gene conversion, a process arising from homology-directed DNA repair during recombination. Gene conversion is the unilateral exchange of genetic material from a donor to an acceptor sequence (Chen et al. 2007). A recombination event is initiated by a double-strand break which is repaired by the cellular machinery using the homologous chromosome as template sequence. If there is a sequence mismatch within the recombination tract, gene conversion may occur (Chen et al. 2007). Mismatches in heteroduplex DNA are repaired by the mismatch-repair machinery (Chen et al. 2007). Importantly, G/C (strong, S, three-hydrogen bonds) to A/T (weak, W, two hydrogen bonds) mismatches can have a resolution bias in favor of S alleles resulting in gBGC, a process that can alter base composition and genetic diversity (Nagylaki 1983; Marais 2003; Duret and Galtier 2009; Mugal et al. 2015). Direct observations of gBGC are restricted to a small number of taxa, such as human (Arbeithuber et al. 2015), baker’s yeast (*Saccharomyces cerevisiae*) (Mancera et al. 2008), collared flycatcher (Smeds et al. 2016), and honey bees (Kawakami et al. 2019). Indirect evidence exists for a wider set of species, including arthropods such as brine shrimp (*Artemia franciscana*) and butterflies from the Hesperidae, Pieridae, and Nymphalidae families (Eyre-Walker 1999; Perry and Ashworth 1999; Meunier and Duret 2004; Muyle et al. 2011; Pessia et al. 2012; Glémin et al. 2015; Galtier et al. 2018).

The strength of gBGC can be measured by the population-scaled parameter *B *=* *4 *N*_e_*b*, where *b *=* ncr* is the conversion bias, which is dependent on the average length of the conversion tract (*n*), the transmission bias (*c*), and the recombination rate per site per generation (*r*) (Glémin et al. 2015; Mugal et al. 2015). This means that we can expect a stronger impact of gBGC in larger populations and in genomic regions of high recombination. Nagylaki (1983) showed that we can understand gBGC in terms of directional selection, that is, the promotion of one allele over another. This leads to a characteristic derived allele frequency (DAF) spectrum, in which an excess of W→S alleles- and a concomitant lack of S→W alleles, are segregating at high frequencies in the population. Nevertheless, the overall number of S→W polymorphism is expected to be higher in most species because of the widely observed S→W mutation bias, partially caused by the hypermutability of methylated cytosines in the 5′-CpG-3′ dinucleotide context (Lynch 2007). Preventing the fixation of ubiquitous and possibly deleterious S→W mutations have been proposed as one of the ultimate causes for gBGC (Brown and Jiricny 1987; Birdsell 2002; Duret and Galtier 2009). However, although gBGC reduces the mutational load it may also confer a substitutional load by favoring deleterious W→S alleles (Duret and Galtier 2009; Glémin 2010; Mugal et al. 2015). This effect has led some authors to describe gBGC as an “Achilles heel” of the genome (Duret and Galtier 2009; Mugal et al. 2015). Detailed analysis of a larger set of taxonomic groups is needed to understand the prevalence and impact of gBGC. There is also limited knowledge about the variation in the strength of gBGC within and between closely related species (Borges et al. 2019).

Here, we investigate the dynamics of gBGC in butterflies and characterize the effect of gBGC on genetic diversity. We use whole-genome resequencing data from 60 individuals from six populations of three species of wood whites (genus *Leptidea*). Wood whites show distinct karyotype- and demographic differences both within and among species (Dincă et al. 2011; Lukhtanov et al. 2011; Dincă et al. 2013; Lukhtanov et al. 2018; Talla, Johansson, et al. 2019; Talla, Soler, et al. 2019). This includes, *L. sinapis*, which has the greatest intraspecific variation in diploid chromosome number of any animal, from 2*n *=* *57, 58 in southeastern Sweden to 2*n *=* *106–108 in northeastern Spain (Lukhtanov et al. 2018). Our objectives are 3-fold. First, we infer the strength and determinants of gBGC variation among *Leptidea* populations. Second, we investigate the patterns of gBGC and mutation bias across the genome, its determinants, and association with GC content. Third, we detail the effect of gBGC and opposing mutation bias on genetic diversity across a GC gradient and consider the impact of linked selection and GC content itself as determinants of genetic diversity.

## Results

### Samples, Genome, and Population Resequencing Data

The samples and population resequencing data used in this study were originally presented in Talla et al. (2017). In brief, 60 male *Leptidea* butterflies from three species and six populations were analyzed. For *L. sinapis*, 30 individuals were sampled: ten from Kazakhstan (Kaz-sin), ten from Sweden (Swe-sin), and ten from Spain (Spa-sin). Ten *L. reali* were sampled in Spain (Spa-rea) and ten *L. juvernica* per population were collected in Ireland (Ire-juv) and Kazakhastan (Kaz-juv), respectively. Reads from all 60 sampled individuals were mapped to a previously available genome assembly of an inbred, male, Swedish *L. sinapis* (scaffold N50 = 857 kb) (Talla et al. 2017). Detailed information on SNP calling can be found in Talla, Johansson, et al. (2019).

### Patterns of gBGC among Populations and Species

**Fig. 1.**
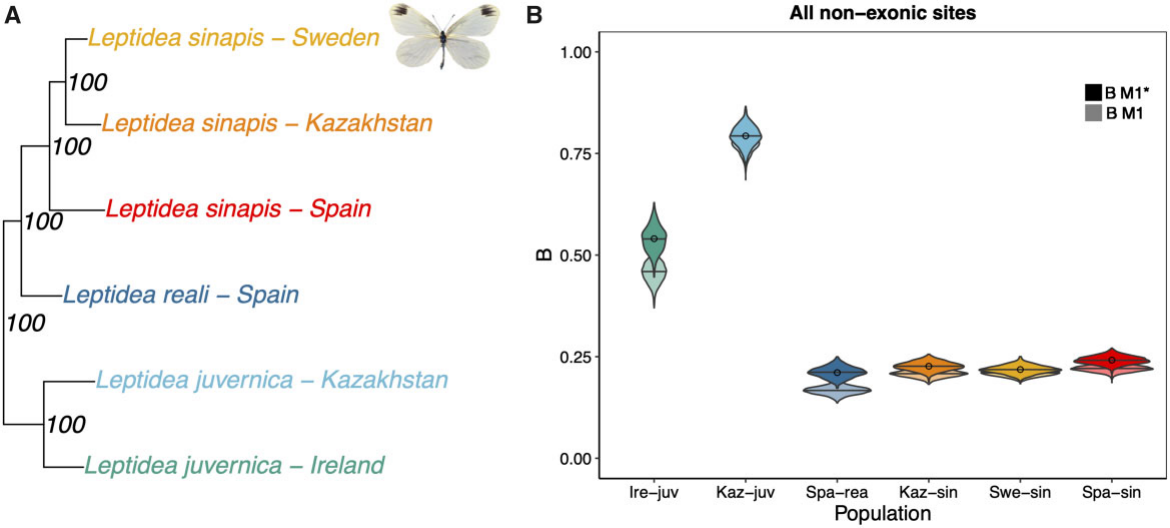
*Leptidea* butterflies show variation in the genome-wide strength of gBGC. (*A*) Phylogeny of the six *Leptidea* populations included in this study. Node values represent support from 100 bootstrap replicates on sites. The phylogeny in (*A*) is based on a subtree from a maximum-likelihood phylogeny used as a starting tree in figure 1 of Talla et al. (2017). A mounted specimen of a *Leptidea sinapis* is shown. (*B*) Estimates of the population-scaled coefficient of gBGC (*B = *4 *N*_e_*b*). Circles represent point estimates from the original DAF spectra using model M1*, bars are mean values of *B* for the 1,000 bootstrap replicates on segregating sites. Overlain and opaque violins are bootstrapped values for model M1* and underlain, transparent violins are estimates for model M1.

**Fig. 2.**
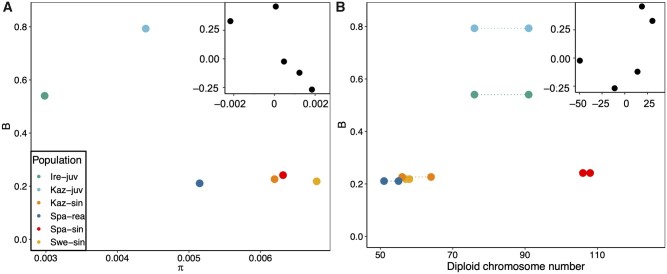
Determinants of variation in the strength of gBGC among populations (*A*) Relationship between π and *B*. (*B*) Relationship between diploid chromosome number and *B* (M1*). Points in (*B*) show the lowest and highest estimate of diploid chromosome number for each population. Insets in (*A*) and (*B*) show phylogenetically independent contrasts of each respective axis variable based on the phylogeny in figure 1*A*. Contrasts for diploid chromosome number were based on midpoint value.

To infer the strength of gBGC in the different *Leptidea* populations (fig. 1*A* and *B*), we calculated separate DAFs per mutation category (GC-conservative/neutral: S→S and W→W, collectively denoted N→N, GC-changing: S→W and W→S) for segregating nonexonic variants. To polarize alleles, we used invariant sites in one or two outgroup populations (“strict” polarization; supplementary table S1, Supplementary Material online). We used the four basic population genetic models developed by Glémin et al. (2015) to obtain maximum likelihood estimates of the intensity of gBGC (*B *=* *4 *N*_e_*b*). Model M0 is a null model with *B* fixed at 0. In contrast, *B* is a free parameter in model M1. To correct for polarization errors, we also used extensions of M0 and M1 (M0* and M1*) with one error parameter included per mutation category. The GC content in the ancestral genome was approximately 0.32. For all populations, the M1 model had a better fit than the M0 model (likelihood-ratio tests [LRT] upper-tailed χ^2^; α = 0.05; df* *=* *1), which indicates that gBGC is a significant evolutionary force in *Leptidea* butterflies (fig. 1*B*)*.* The quantitative results from the M1 and M1* models were overall congruent, and M1* had a better fit for all populations except Swe-sin (LRT upper-tailed χ^2^; α = 0.05; df* *=* *3). When taking all nonexonic sites into consideration and applying model M1*, Spa-rea and Swe-sin had the lowest *B* (0.21), followed by Kaz-sin (*B *=* *0.22). Spa-sin, the population with the largest number of chromosomes (fig. 2*B*), had a marginally higher *B* (0.24) compared with the other *L. sinapis* populations. All these estimates were lower than Irish- (Ire-juv) and Kazakhstani (Kaz-juv) *L. juvernica* with *B *=* *0.54 and *B *=* *0.79, respectively (supplementary table S2, Supplementary Material online).

We tried an alternative more “liberal” polarization (only two outgroup individuals, see Materials and Methods) to test the impact of the polarization scheme on the estimates from the gBGC model. The results were qualitatively similar but the polarization error rates were inflated compared with the “stricter” polarization scheme (supplementary table S2 and text S1, Supplementary Material online). Thus, we used the “strict” polarization scheme for subsequent analyses unless otherwise stated. We also tested the impact of including and excluding ancestral CpG-prone sites as they may influence the estimation of the S→W mutation bias (λ) and *B* (supplementary text S1, Supplementary Material online). All populations except Kaz-juv had the highest estimate of λ at ancestral CpG-prone sites, followed by all nonexonic sites and lowest when excluding ancestral CpG sites (supplementary table S2, Supplementary Material online). This difference could be caused by hypermutagenic methylated cytosines but the level of DNA methylation observed in Lepidopteran taxa is low (Jones et al. 2018). However, the difference when excluding- and including CpG-prone sites was small (<0.25) and consequently, we used all nonexonic sites in subsequent analyses.

### Determinants of gBGC Intensity Variation among Populations and Species

The strength of gBGC is dependent on *N*_e_ and the conversion bias *b *=* ncr*. Given that transmission bias, *c*, and conversion tract length, *n*, require sequencing of pedigrees, we here focus on variation in genome-wide recombination rate, *r* to assess variation in *b.* To understand the relative importance of *N*_e_ and *r*, we correlated *B* with π (as a proxy for *N*_e_) and diploid chromosome number (as a proxy for genome-wide recombination rate) (Kaback et al. 1992; Stapley et al. 2017). Neither genetic diversity, (π*; P *≈* *0.13, adjusted *R*^2^* *≈* *0.45), nor diploid chromosome number (*P *≈* *0.35, *R*^2^* *≈* *0.05), significantly predicted variation in *B* among species in phylogenetically independent contrasts (see insets in fig. 2*A* and *B*). Since Spanish *L. sinapis* likely experienced massive chromosomal fission events recently (Lukhtanov et al. 2011; Talla, Johansson, et al. 2019; Lukhtanov et al. 2020), it is possible that *B* is below its equilibrium value in this population. Excluding Spa-sin yielded a positive relationship between chromosome number and the intensity of gBGC, though above a significance threshold of 0.05 (*P* ≈ 0.07, *R*^2^* *≈* *0.79).

### Level of Mutation Bias Varies among *Leptidea* Species

The GC content is determined by the relative fixation of S→W and W→S mutations (Sueoka 1962), which is governed by the balance of a mutation bias from S→W over W→S, and a fixation bias from W→S over S→W. The latter may be caused by gBGC only, but may also be observed at synonymous sites due to selection for preferred codons (Galtier et al. 2018). Protein coding genes make up only 3.7% of the *L. sinapis* genome (Talla, Soler, et al. 2019) and potential selection on codon usage will hence only affect genome-wide base composition marginally in this species. Using the DAF spectra of different mutation classes allows not only estimation of *B*, but also the mutation bias, λ (Muyle et al. 2011; Glémin et al. 2015). We found that λ (estimated from model M1*) varied from 2.94 (e.g., Spa-sin) to 4.09 (Kaz-juv) (supplementary table S2, Supplementary Material online). This means that the S→W mutation rate is on average 3 to 4 times higher than the W→S mutation rate in *Leptidea* butterflies. Applying the M1 model gave similar results. It is possible that the polarization scheme which only allowed private alleles for the *L. juvernica* populations, contributed to their high value of λ*.* To test this, we used the aforementioned “liberal” polarization. The resulting λ were approximately 3.5 and 3 for Kaz-juv and Ire-juv, respectively, and approximately 3 for the *L. reali* and *L. sinapis* populations, with only minor differences in λ between the M1 and M1* models for all populations (supplementary table S2, Supplementary Material online). This indicates that the “strict” polarization scheme shape the DAF spectra of the *L. juvernica* populations in a way unaccountable for by the demographic *r_i_* parameters of the model. However, this aspect of the polarization scheme alone cannot explain the higher λ observed in Kaz-juv compared with the other populations (see supplementary text S1, Supplementary Material online, for further discussion).

### Patterns and Determinants of gBGC and GC Content across the Genome

**Fig. 3.**
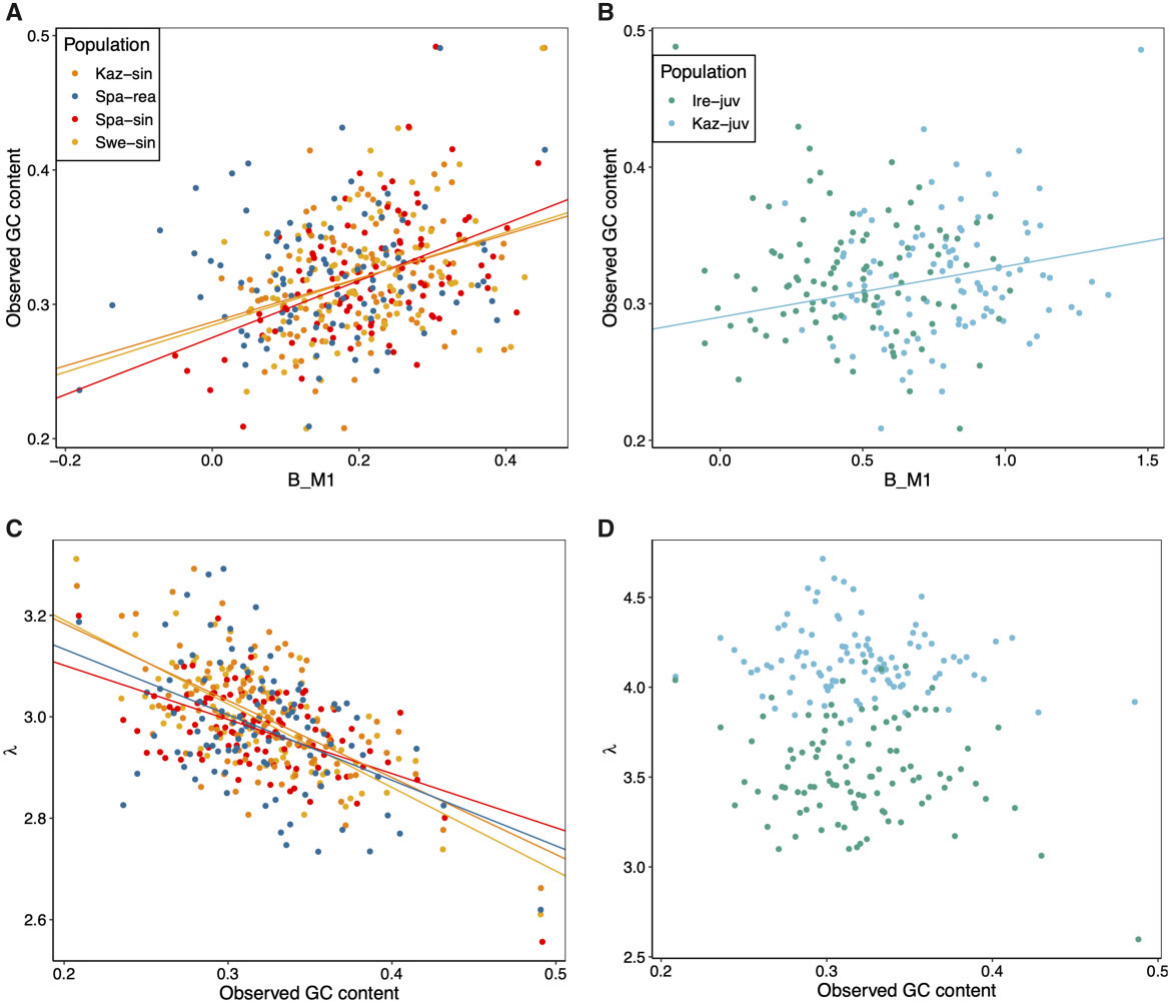
Relationship between *B*, λ, and observed GC content in the ancestral genome. (*A*) Association between *B* and observed GC content in the ancestral genome for the *L. sinapis*–*L. reali* clade, and (*B*) for the *L. juvernica* populations. Higher GC content was significantly consistent with greater *B* in all populations except Spa-rea and Ire-juv. (*C*) Relationship between λ and GC content was negative for all populations in the *L. sinapis*–*L. reali* clade. (*D*) Shows the same as (*C*) but for the *L. juvernica* populations. Neither Kaz-juv nor Ire-juv showed significant associations between λ and GC content. Lines in plots represent significant linear regressions performed separately per population between the *X*- and *Y* variables.

To understand the effects of gBGC throughout the genome, we partitioned the polarized SNPs into centiles based on their local (1 kb) GC content in the ancestral genome. The number of SNPs in each centile ranged from 2,661 in Ire-juv to 21,140 in Spa-sin (supplementary table S1, Supplementary Material online). The models were compared using LRTs on the average difference of all centiles between the reduced (M0) and full (M1) model and between the models excluding (M1) or including (M1*) polarization error parameters. M0 could not be rejected in favor of M1 for both Ire-juv and Spa-rea. It is possible that the lower number of SNPs per GC centile in these populations increases variance and thus reduces the fit of the M1 model, especially for Spa-rea which had the lowest *B* (fig. 1*B*). However, both of these populations had a genome-wide significant influence of gBGC, and will still be considered in the following analyses. For all populations, M1* was not significantly better than M1, indicating either a lack of power for M1* or that the polarization error was negligible. The strength of gBGC (*B *=* *4 *N*_e_*b*) varied across GC centiles for all populations with Swe-sin and Kaz-sin showing the lowest standard error of the mean (0.009, table 1; fig. 3*A* and *B*) and Ire-juv the highest (0.026). Because Ire-juv had the lowest number of SNPs per centile, it is hard to disentangle sample—from biological variance but we note that Kaz-juv showed a similar standard error (0.025). The average value was overall congruent with what we observed in the analysis among populations (supplementary table S2, Supplementary Material online). We saw similar standard errors for the S→W mutation bias, λ (table 1; fig. 3*C* and *D*).

**Table 1:** Estimates of λ, *B*, and Relevant Measures of GC Content

Population	λ	*B*	GC 1/(1* *+* *λ)	GC 1/(1* *+* *λe^−^^*B*^)	GC π_max_	GC CDS_min_
Swe-sin	2.99 ± 0.010	0.21 ± 0.009	0.25	0.29	0.27	0.35
Spa-sin	2.97 ± 0.008	0.21 ± 0.010	0.25	0.29	0.31	0.34
Kaz-sin	3.00 ± 0.011	0.20 ± 0.009	0.25	0.29	0.28	0.34
Kaz-juv	4.15 ± 0.019	0.79 ± 0.025	0.19	0.35	0.29	0.34
Ire-juv	3.54 ± 0.027	0.47 ± 0.026	0.22	0.31	0.29	0.34
Spa-rea	2.98 ± 0.012	0.16 ± 0.011	0.25	0.28	0.31	0.34

Note.—Population-specific averages across GC centiles of λ, *B*, equilibrium GC content under mutational equilibrium alone, GC**(**1/[1* *+* *λ]), and when taking *B* into account GC(1/[1* *+* *λe^−^^*B*^]), and the observed GC content in the ancestral genome for the centile with the highest average pairwise difference GC(π_max_) and lowest density of coding sequence (GC CDS_min_). We also show standard error of the mean for λ and *B*.

To investigate the impact of variation in *N*_e_ across the genome, we used genetic diversity, as a proxy for *N*_e_ and predictor of *B*, in separate linear regressions for each population (supplementary fig. S1*A*, Supplementary Material online)*.* Swe-sin and Kaz-sin showed significant negative relationships (*P < *0.05), but limited variance explained (*R*^2^* ≈ *0.1 for both). The regressions were insignificant (*P > *0.05) for the other populations (supplementary fig. S1*A*, Supplementary Material online). Overall these results suggest that variation among centiles in *B* could be dominated by differences in conversion bias, *b*, instead of variation in *N*_e_. An observation that supported this conclusion is that *B* significantly (*P < *0.05; *R*^2^: 4–22%) predicted GC content in four out of six populations (fig. 3*A* and *B*). Here, GC content may serve as a proxy for recombination rate, assuming that differences in GC content have been caused by historically higher rates of recombination and thus stronger *B.* That two populations lacked a relationship with GC content may be explained partly by a lack of power for Ire-juv, which had the lowest number of SNPs per centile, whereas this explanation is less likely for Spa-rea. Nevertheless, for a majority of the populations considered here we saw a relationship between GC content in the ancestral genome and *B*, indicating that gBGC has been influencing the evolution of GC content.

The mutation bias was significantly (*P < *0.05, separate linear regression per population) negatively associated with observed GC content in the ancestral genome for all populations except Ire-juv and Kaz-juv (fig. 3*C* and D). To investigate if there was an association between λ and *B*, we performed separate linear regressions per population predicting λ with *B*. Higher estimates of λ across the genomes were consistent with larger values of *B* for all populations (*P < *0.05) except Spa-sin and Swe-sin (supplementary fig. S1*B*, Supplementary Material online). This indicates an inability of the model to separately estimate these parameters or increased *B* in regions more prone to S→W mutations. The former explanation is unlikely given that the most common sign was negative in the regressions between λ and GC content.

### Mutation Bias and gBGC Influence the Evolution of GC Content

**Fig. 4.**
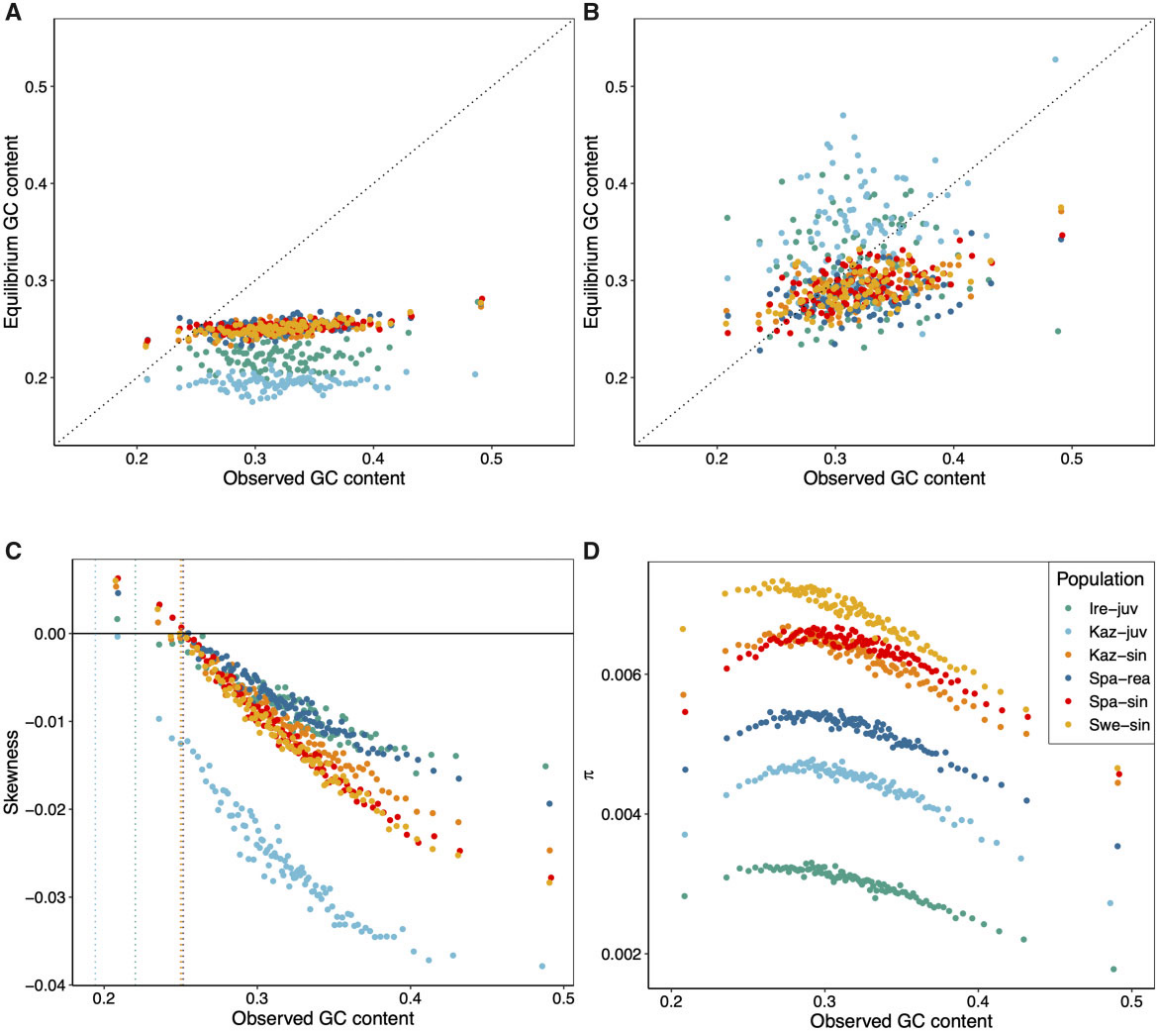
Observed GC content, equilibrium GC content and their association with λ, *B*, and genetic diversity (π). (*A*) Observed GC content compared with equilibrium GC content determined by mutation bias (λ) alone. (*B*) Observed GC content compared with equilibrium GC content when accounting for gBGC. Dotted lines in (*A*) and (*B*) represent *x* = *y*. (*C*) The skewness of the folded SFS shows the strong S→W bias in the segregating variation which increases with observed GC content in the ancestral genome. Extrapolating from the distribution of skewness values onto the *y* = 0 line serves as a validation of the estimated λ. Dotted vertical lines represent the GC equilibrium under mutation bias alone, 1/(1* *+* *λ), for each population. (*D*) The association between genetic diversity (π) and observed GC content. Points in all panels represent GC centiles.

The equilibrium GC content in the presence of a S→W mutation bias, but in the absence of gBGC, can be calculated as 1/(1* *+* *λ) (Sueoka 1962). The observed mean GC content was 0.32 for all populations, which is higher than expected under mutational equilibrium alone across almost the entire genome for all populations (fig. 4*A*). When accounting for gBGC (1/[1* *+* *λe^−^^*B*^]) (Li et al. 1987; Bulmer 1991; Muyle et al. 2011), the observed mean GC content was higher than the predicted equilibrium GC content in all populations except Kaz-juv (table 1 and fig. 4*B*). This means that gBGC in general is not strong enough to prevent GC content from decreasing in all the considered *Leptidea* populations except Kaz-juv.

Segregating variants hold information on the evolution of base composition. GC content will decrease if more S→W than W→S mutations reach fixation and vice versa. We can explore the fate of segregating variants by investigating the skewness of the folded site-frequency spectrum (SFS) (fig. 4*C*) (Glémin et al. 2015). GC content is at equilibrium if skewness equals zero, evolves to higher GC content if the skew is positive, and decreases if it is negative. As expected from the relationship between observed and equilibrium GC content (fig. 4*A* and *B*), most of the centiles in all populations had a negative skew, which shows that GC content is decreasing in the *Leptidea* genomes (fig. 4*C*).

### Pinnacle of Genetic Diversity Close to GC Equilibrium

We found a nonmonotonic relationship between GC content and π (fig. 4*D*). The highest genetic diversity was observed close to the predicted genome-wide GC equilibrium, with diversity decreasing in both directions away from equilibrium GC content (fig. 4*D*). To test if this pattern could result from differential read coverage, we calculated the average read count per base pair in each GC centile per individual (supplementary fig. S2, Supplementary Material online). Read coverage was generally even across most of the GC gradient except for two regions around 31% and 35% GC where the *L. juvernica* populations show a signal consistent with duplications compared with the *L. sinapis* reference genome. In addition, the centile with the greatest GC content showed high coverage in all populations. This is expected given the PCR bias against high and low GC regions in Illumina sequencing (Browne et al. 2020). With the exception of *L. reali*, the GC content at the centile with the highest π, GC(π_max_), was at a level between the GC equilibrium defined by λ alone, GC(1/[1* *+* *λ]), and equilibrium when accounting for both λ and *B*, GC(1/[1* *+* *λe^−^^*B*^]). GC(π_max_) was lower for all populations than the GC content of the centile with the lowest density of coding sequence, GC(CDS_min_).

### The Role of gBGC and Mutation Bias in Shaping Genetic Diversity

**Fig. 5.**
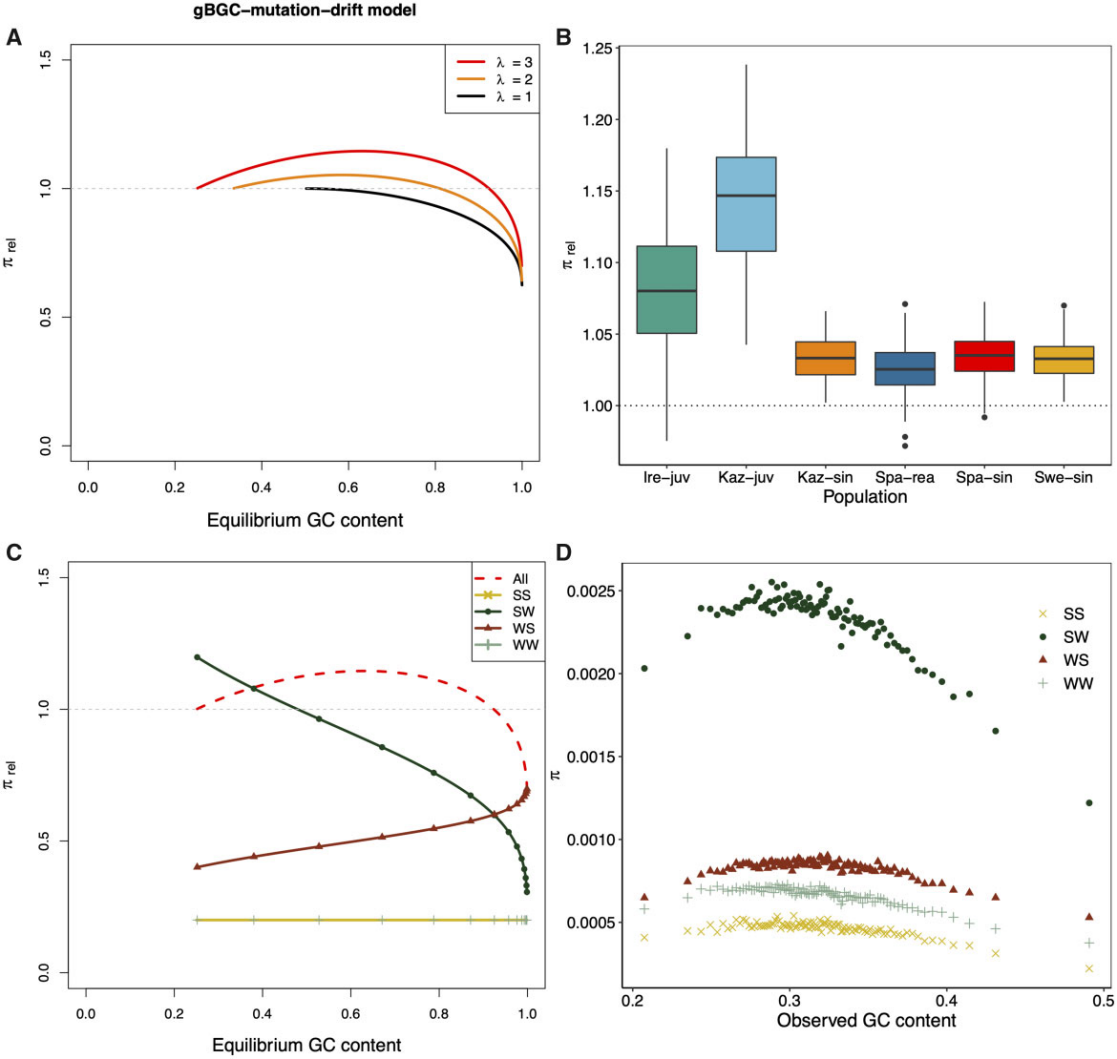
A model for genetic diversity under gBGC–mutation–drift equilibrium, predicted π_rel_ per population and π per mutation category. (*A*) Genetic diversity relative to neutral (*B *=* *0) across equilibrium GC content determined by *B* and λ. Lines begin at *B = *0 and end at *B *=* *8. The mutation bias is held constant. (*B*) Genetic diversity values predicted from the gBGC–mutation–drift equilibrium model using output from the inference of gBGC. Most of the genomes for each population have values of *B* and λ such that their relative strength boosts the long-term genetic diversity compared with *B *=* *0. The lower and upper limits of the box correspond to the first and third quartiles. Upper and lower whiskers extend from the top- and bottom box limits to the largest/smallest value at maximum 1.5 times the interquartile range. (*C*) Components of the gBGC mutation drift model. Only results from λ* *=* *3 are shown. The separate mutation categories were standardized by mutational opportunity, whereas “All” was standardized as in (*A*). The genetic diversity is here assumed to be equal for N→N and W→S mutations (θ_N_/θ_WS_* *=* *1). (*D*) Genetic diversity in Swedish *L. sinapis* measured by average pairwise differences (π) across genomic GC content for all four mutation categories: S→S (SS), S→W (SW), W→S (WS), W→W (WW). The other populations are shown in supplementary figure S4, Supplementary Material online.

Since gBGC mimics selection, the genetic diversity is directly dependent on the interaction between the strength of gBGC and the potential of an opposing mutation bias (McVean and Charlesworth 1999). To understand how gBGC contributes to genetic diversity in *Leptidea*, we estimated the effects of gBGC and opposing mutation bias on genetic diversity by modeling the effect of *B* on the SFS (McVean and Charlesworth 1999). In the model, gBGC elevates the relative genetic diversity (π_rel_) in the presence of an opposing mutation bias (λ* *>* *1) by increasing the equilibrium GC content compared with the case when gBGC is absent (*B *=* *0). This allows for a greater influx of mutations as long as λ* *>* *1 (fig. 5*A*). In *Leptidea*, genetic diversity (π) showed a nonmonotonic relationship along the GC range (fig. 4*D*). In contrast, given values of λ around 3 and above, relevant for *Leptidea*, the model assuming gBGC–mutation–drift equilibrium (GMD) predicts a monotonic increase of π in the 0.2–0.5 GC range (fig. 5*A*). Using the output from the gBGC inference, we could predict π_rel_ values for each GC centile and population from the GMD model (fig. 5*B*). The results showed that gBGC and mutation bias has the potential to elevate π compared with *B *=* *0, by an average of 2.6% in Spa-rea, 3.3% in Swe-sin and Kaz-sin, 3.5% in Spa-sin, 8% in Ire-juv, and 14.7% in Kaz-juv. According to the GMD model, this means that at GC equilibrium, gBGC will promote genetic diversity in *Leptidea* butterflies.

We can decompose the GMD model into four spectra standardized by their respective mutational opportunity (fig. 5*C*) to mimic the empirical data (fig. 5*D*). For example, the S→W category is standardized by equilibrium GC content. The four spectra include the GC-conservative/neutral spectra (W→W and S→S) and the GC-changing spectra (W→S and S→W) (fig. 5*C*). The contribution of GC-conservative mutation categories to π is unaffected by equilibrium GC content. In contrast, the influence of S→W on the SFS decreases as *B* increases, and vice versa for W→S over the GC range. We also tested the robustness of the model to variation in of λ and *B* by drawing values of both parameters from normal distributions in which the standard deviation was determined from observed values (GC centile analysis) from Swedish *L. sinapis* (0.1 and 0.09 for λ and *B*, respectively, supplementary fig. S3, Supplementary Material online). Although some variation is evident (especially for the S→W mutation class because of the λ in the numerator, see Materials and Methods), the overall qualitative pattern is unaffected by the estimation error observed in the empirical data.

To understand the role gBGC plays in the variation of π with GC in *Leptidea*, we investigated the properties of the DAF spectra separately for all four mutation categories mentioned above. A majority of the segregating sites were GC-changing and S→W contributed most to π across all centiles (Swe-sin: fig. 5*D*, Others: supplementary fig. S4, Supplementary Material online). All mutation classes showed a qualitatively negative quadratic relationship between π and GC content (fig. 5*D* and supplementary fig. S4, Supplementary Material online). We therefore suggest that the roughly negative quadratic curves of π over GC content are to some degree shaped by factors shared among mutation classes. This means that forces other than gBGC are the main determinants of the relationship between GC content and diversity (cf. fig. 5*C* and *D*). In addition, we note that observed differences between W→W and S→S diversity ask for a refined GMD model beyond binary states W and S.

### The Effects of Linked Selection and GC Content on Genetic Diversity

**Fig. 6.**
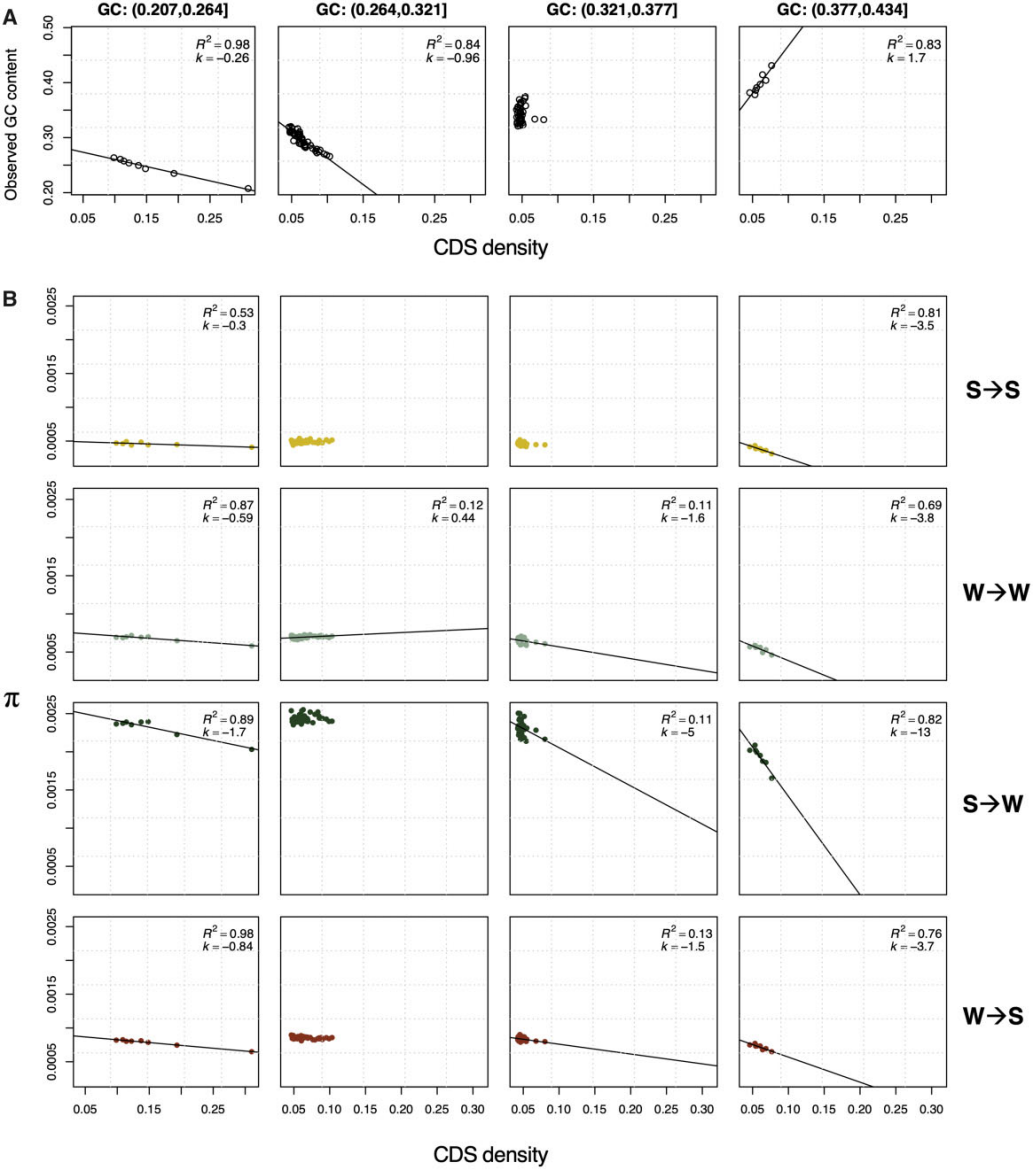
Relationship between π, CDS density, and GC content. (*A*) shows the relationship between CDS density and GC content for Swe-sin in four nonoverlapping equidistant intervals of GC content. (*B*) instead shows the relationship between π and CDS density in the same bins separately for: S→S, W→W, S→W, and W→S mutations. The fifth GC content bin is not shown because it includes only one centile. See supplementary figure S5, Supplementary Material online, for the other populations. *R*^2^* = *proportion of variation explained, *k *=* *slope of regression (times 10^3^ for readability in *B*). GC bins 1–4 shown left to right. Mutation categories from top to bottom row: S→S, W→W, S→W, and W→S.

Having rejected gBGC as a main contributor to π along the GC gradient warrants the question: can the pattern be explained by reductions in diversity caused by linked selection? Linked selection has previously been shown to affect genetic diversity in butterfly genomes (Martin et al. 2016; Talla, Soler, et al. 2019). Selection affecting linked sites will reduce genetic diversity unequally across the genome depending on the density of targets of selection and the rate of recombination. In agreement with this, density of coding sequence (CDS density), which can be used as a proxy for the intensity of linked selection in general but background selection in particular (Lohmueller et al. 2011), was larger where π was lower (fig. 6 and supplementary fig. S5, Supplementary Material online).

In addition, regional variation in mutation rate will also contribute to a heterogenous diversity landscape. We here suggest that GC content influences mutation rate for three reasons: 1) π varies conspicuously with GC content (fig. 4*D*), 2) the S→W mutation bias appears to be affected by GC content (fig. 3*C*), and, 3) GC content has been shown to be a major determinant of the mutation rate at CpG sites in humans (Fryxell and Moon 2005; Tyekucheva et al. 2008; Schaibley et al. 2013). Since guanine and cytosine are bound by three hydrogen bonds, one more than for adenine and thymine, it is believed that a higher local GC content reduces the formation of transient single-stranded states (Inman 1966). Cytosine deamination, which leads to C/G→T/A mutations, occurs at a higher rate in single-stranded DNA (Frederico et al. 1993). Thus a higher GC content appears to reduce CpG mutation rates on a local scale of approximately 2 kb (Elango et al. 2008). Mutation rate variation determined by local GC content outside the CpG context is less studied but negative correlations have been observed for most mutation classes in humans (Schaibley et al. 2013).

To understand the relative contribution of GC content and CDS density on variation in π, we first used quadratic regression analyses separately for each population and mutation category. We started with a model including both linear and quadratic terms for GC content and CDS density as well as an interaction term and performed stepwise reduction of insignificant predictor terms (supplementary table S4, Supplementary Material online). In general, the best fitting model for all populations and categories included linear terms of GC content and CDS density, both showing negative relationships with π, where GC content was a stronger predictor in most cases (higher absolute value of *t*).

Since GC content and CDS density were correlated with each other, we separated the data into bins (fig. 6*A*). The GC centiles were placed in five bins of equidistant GC content and variants were separated by mutation category (fig. 6 and supplementary fig. S5, Supplementary Material online). This allows an investigation of the impact of CDS density on π while keeping the GC range constant, and thereby disentangle the relative contribution of GC content and CDS density on variation in π. The fifth bin was not considered as it included only a single centile with the highest GC content. First, we studied the association between GC content and CDS density (fig. 6*A*). GC content was negatively associated with CDS density in bins 1 and 2, whereas bin 3 showed no relationship and bin 4 a positive correlation (fig. 6*A*). Second, we considered the relationship between π and CDS density for all mutation categories. Here, the general trend was negative, across GC bins, populations, and mutation categories. In addition, the slopes got more negative with increasing GC content (fig. 6*B* and supplementary fig. S5, Supplementary Material online).

For the GC-neutral mutation categories, we observed the steepest negative slope when CDS density and GC content had a positive relationship (bin 4, fig. 6*A* and supplementary fig. S5, Supplementary Material online). This may be caused by a joint effect of higher local GC content and CDS density contributing to a strong reduction in genetic diversity (fig. 6*A* and *B*). Despite a similar spread in CDS density, most populations showed fewer significant trends for bin 2. For Swe-sin, the W→W mutation category even showed a positive slope (fig. 6*B*). This is possibly a result of the negative relationship between GC content and CDS density causing an antagonistic response on diversity. When only GC content varied, π was also reduced for some but not all mutation categories and populations (bin 3, fig. 6 and supplementary fig. S5, Supplementary Material online). When CDS density and GC content had a negative relationship, the slope was shallow but lower π was still consistent with a higher proportion of coding sequence (bin 1, fig. 6). From these results, we conclude that both GC content itself and linked selection affect diversity across the genome in *Leptidea* butterflies.

For the GC-changing mutation categories, we observed some evidence that gBGC has affected genetic diversity (fig. 6*B* and supplementary fig. S5, Supplementary Material online). The decomposed GMD model—with separate categories standardized for mutational opportunity—predicts that π will increase and decrease monotonically with GC content for W→S and S→W mutations, respectively (fig. 5*C*). Our results supported this conclusion for the S→W category which showed a more pronounced negative slope compared with the GC-neutral mutation categories (fig. 6*B* and supplementary fig. S5, Supplementary Material online). However, this pattern is also expected from the relationship between λ and GC content (fig. 3*C*). The W→S mutation category showed slopes comparable with the GC-neutral categories which means that it did not follow the expectation of the GMD model. Linked selection could interact with the distortion of the S→W and W→S DAF spectra caused by gBGC, which would constitute an indirect effect on π by gBGC. An argument against an indirect effect is that linked selection would be weaker or diminish where recombination is the highest, which most likely occur at greater GC content where *B* is stronger (see Discussion, fig. 3*A* and *B*) (Pouyet et al. 2018).

## Discussion

### The Intensity of gBGC Varies Widely among Species

In this study, we used whole-genome resequencing data from several populations of *Leptidea* butterflies to estimate gBGC and investigate its impact on rates and patterns of molecular evolution. Our data support previous observations that gBGC is present in butterflies (Galtier et al. 2018). The genome-wide level of gBGC (*B*) varied from 0.17 to 0.80 among the investigated *Leptidea* populations*.* In general, *L. juvernica* populations had levels of *B* in line with previous estimates of gBGC in butterflies (0.69–1.16; Galtier et al. 2018), whereas the other species had lower *B*, more in agreement with what has been observed in humans (0.38) (Glémin et al. 2015).

### Determinants of gBGC Variation in Animals

Regression analysis suggested that the overall strength of gBGC among the *Leptidea* butterflies may depend more on interspecific variation in genome-wide recombination rate rather than differences in *N*_e_. Galtier et al. (2018) also showed a lack of correlation between *B* and longevity or propagule size (used as proxies for *N*_e_), across a wide sample of animals. We observed that chromosome number (a proxy for genome-wide recombination rate) was positively associated with *B* after excluding Spa-sin, which has recently experienced a change in karyotype. Galtier et al. (2018) suggested that *B* may vary among species due to interspecific differences in transmission bias, *c*. This observation was supported by a study on honey bees (*Apis mellifera*) showing a substantial variation in transmission bias at noncrossover gene conversion events (0.10–0.15) among different subspecies (Kawakami et al. 2019). Analyses of noncrossover gene conversion tracts in mice and humans showed that only conversion tracts including a single SNP were GC-biased (Li et al. 2019). By contrast, in yeast, the SNP closest to the end of a conversion tract determines the direction of conversion for all SNPs in a tract (Lesecque et al. 2013). Both these studies suggest that the impact of conversion tract length may be more complex than the multiplicative effect on conversion bias assumed in the *b *=* ncr* equation. The relative importance of recombination rate, transmission bias, and conversion tract length, in divergence of *b* among populations and species remains to be elucidated.

### Butterfly Population Genomics in Light of gBGC

Linkage maps for butterflies with high enough resolution to establish whether or not recombination is organized in hotspots are currently lacking (Davey et al. 2016; Davey et al. 2017; Halldorsson et al. 2019). Nevertheless, recombination varies marginally (2-fold) between—but substantially within chromosomes in two species of the *Heliconius* genus (Davey et al. 2017). Related to this, chromosome length is negatively correlated to both recombination rate and GC content in *H. melpomene* (Martin et al. 2016; Davey et al. 2017; Martin et al. 2019), which is a pattern typical of gBGC (Pessia et al. 2012). Shorter chromosomes experience on an average more recombination events due to the observation of at least one crossover per chromosome (or chromosome arm) per meiosis in most animals (excluding, e.g., butterfly females and *Drosophila* males) (Baker et al. 1976; Kaback et al. 1992). This, in turn, leads to a stronger signature of GC-biased gene conversion on shorter chromosomes. The higher GC content at 4-fold degenerate (4 D) sites on shorter chromosomes in *H. melpomene* was interpreted to be a consequence of stronger codon usage bias on short chromosomes (Martin et al. 2016). An alternative explanation is that the higher recombination rate per base pair observed on smaller chromosomes leads to an increased intensity of gBGC and consequently a greater GC content. Galtier et al. (2018) showed significant positive correlations (*r *=* *0.18–0.39) between GC content of the untranslated region and the third codon position in genes of three butterflies. This supports the conclusion that gBGC and possibly variation in mutation bias across the genome, affects codon usage evolution in butterflies. The degree of mutation bias in *H. melpomene* is unknown (as far as we know), but a λ* *≈* *3 is possible given that *H. melpomene* has a genome-wide GC content of 32.8% (Challis et al. 2017), which is similar to the ancestral *Leptidea* genome and the *L. sinapis* reference assembly (Talla et al. 2017; Talla, Johansson, et al. 2019). We conclude that assessment of natural selection using sequence data should also include disentangling the effects of potential confounding factors like gBGC, especially in taxa where this mechanism is prevalent (Bolívar et al. 2016; Pouyet et al. 2018).

### GC-Biased Gene Conversion, Mutation Bias, and Genetic Diversity

Many studies have in the recent decades investigated the association between genetic diversity and recombination rate and have in general found a positive relationship (Begun and Aquadro 1992; Nachman 1997; Kraft et al. 1998; Cutter and Payseur 2003; Lohmueller et al. 2011; Langley et al. 2012; Cutter and Payseur 2013; Mugal et al. 2013; Corbett-Detig et al. 2015; Wallberg et al. 2015; Martin et al. 2016; Pouyet et al. 2018; Rettelbach et al. 2019; Talla, Soler, et al. 2019; Castellano et al. 2020). Somewhat later, debates on the determinants of so-called GC isochores in mammalian genomes gave rise to much research on the impact of gBGC on sequence evolution (Eyre-Walker 1999; Eyre-Walker and Hurst 2001; Meunier and Duret 2004; reviewed in Duret and Galtier [2009]). In this study, we emphasize that gBGC and the widespread opposing mutation bias may also influence variation in genetic diversity across the genome. This can be considered as an extended neutral null model to which the importance of selective forces can be compared.

Several empirical studies have noted the impact of gBGC on genetic diversity. Castellano et al. (2020) observed that the π of GC-changing mutations had a stronger positive correlation with recombination than GC-conservative mutations. Pouyet et al. (2018) observed that in genomic regions with sufficiently high recombination to escape background selection, GC-neutral mutations were evolving neutrally, whereas S→W mutations were disfavored and W→S mutations favored. This illustrates an important point that genomic regions where the diversity-reducing effects of background selection may be weak or absent, are the same regions in which gBGC affects the SFS the most. Consequently, we suggest that future studies on the impact of linked selection also consider the impact of gBGC. A simple solution would for example be to compare observed data with predictions from the GMD model and consider GC-neutral and GC-changing mutations separately (Castellano et al. 2020).

The impact of gBGC on genetic diversity is dependent on the evolutionary timescale considered. For segregating variants, gBGC can only decrease diversity. If we also consider substitutions and model the evolution over longer timescales, gBGC may indirectly increase genetic diversity. In the GMD equilibrium model, gBGC raises genetic diversity indirectly by increasing GC content, which in turn allows greater mutational opportunity for S→W mutations. This can only be achieved when there is a S→W mutation bias greater than one and the intensity of gBGC is not too strong. Under identical conditions, gBGC may produce a positive correlation between recombination rate and genetic diversity through an increase in GC content. The impact of this effect will depend on the relative proportion of GC-neutral- and GC-changing variants. In the GMD model, the diversity of GC-neutral variants is unaffected by GC content. Although this is a reasonable null model, it is also a simplistic view in light of the diversity-reducing effects on GC-neutral variants imposed by high GC content observed in our study. GC-neutral variants are only independent of gBGC on the timescale of segregating variation. Over longer timescales gBGC and mutation bias will cause GC-content to evolve towards an equilibrium which may or may not be conducive for GC-neutral mutations.

### Determinants of Genetic Diversity across the Genome

Identifying determinants of genetic diversity and evaluating their relative importance remains a challenging task. First, we usually lack information on the relationship between GC content and mutation rate due to the sizable sequencing effort required to establish reliable estimates (Messer 2009). Divergence at synonymous sites has been used as a proxy for mutation rate (Martin et al. 2016; Talla, Soler, et al. 2019), but synonymous divergence is a biased estimator of mutation rate in systems where *B ≠ *0 (Bolívar et al. 2016). In model organisms, such as humans, it has become feasible to study mutation rates using singletons in massive samples (>14,000 individuals; Schaibley et al. 2013), or through large-scale sequencing of trios (Jónsson et al. 2017). Second, the predictor variables of interest are often correlated (e.g., GC content and recombination rate in the presence of gBGC) which complicates interpretation for conventional multiple linear regression approaches (Talla, Soler, et al. 2019). A solution to this problem has been to use principal component regression (PCR) in which the PCs of predictor variables are used as regressors (Mugal et al. 2013; Martin et al. 2016; Dutoit et al. 2017). Using this method, Dutoit et al. (2017) found that the PC which explained most variation of π among 200 kb windows in the collared flycatcher genome was mainly composed of a negative correlation with GC. Martin et al. (2016) considered 4 D sites in *H. melpomene* and found that GC content was less important than gene density. It is likely that synonymous variants show greater impact of background selection compared with nonexonic variants, given the tight linkage between synonymous sites and nonsynonymous sites putatively under (purifying) selection. Instead of PCR, we opted for an alternative approach in which the quadratic relationship between GC content and CDS density was binned into separate categories showing differential correlations. For example, in one bin, GC content and CDS density showed a clear negative correlation (*k = *−0.96, bin 2, fig. 6*A*), and in this bin, the genetic diversity was almost invariant. This would suggest that the concordance between the GC(π_max_) and GC(1/[1* *+* *λe^−^^*B*^]) is a byproduct of the interaction between mutation and linked selection. However, given that GC(1/[1* *+* *λe^−^^*B*^]) is close to GC(π_max_), the balance between gBGC and opposing mutation bias in these populations is driving GC content to values which favor diversity. In addition, by investigating the GC-neutral and GC-changing mutation categories separately, we could to some extent distinguish the effects of linked selection, from the effects of gBGC. The effects of GC content in itself is harder to separate from gBGC as it may differ between mutation categories resulting in patterns congruent with predictions from the GMD model. For example, the S→W category showed stronger negative slopes across bins (especially evident in bin 4, fig. 6*B*) compared with the GC-neutral categories in line with both the GMD model and the observed negative relationship between λ and GC content for a majority of the populations (fig. 3*C*). Nevertheless, the effects of gBGC and mutation bias on π within the GMD model should be interpreted with caution given that it describes π at GC content equilibrium, whereas in reality, many centiles are some distance away from equilibrium.

## Conclusion

In this study, we highlight that gBGC is a pervasive force, influencing rates and patterns of molecular evolution both among and across the genomes of *Leptidea* butterflies. We further emphasize that gBGC shapes genetic diversity and may—through fixation of W→S mutations—lead to a concomitant increase in diversity if opposed by a S→W mutation bias. This means that positive correlations between genetic diversity and recombination do not necessarily imply that selection is affecting diversity in the genome. Especially if the recombination rate is correlated with GC content, a pattern typical of gBGC. Here, we reject gBGC as a main determinant of diversity in *Leptidea* butterflies but recognizes its impact on diversity along with linked selection and GC content. Our model of how mutation bias and gBGC affect segregating variation provides a part of the puzzle linking the evolution of GC content to genetic diversity.

## Materials and Methods

### Data Set

We used 60 male *Leptidea* butterflies from three species and six populations ranging from Kazakhstan in the east to Spain in the west. Further information on parameters used for genotype calling can be found in Talla, Johansson, et al. (2019). Chromosome numbers for each population (if available) or species were obtained from the literature (Dincă et al. 2011; Lukhtanov et al. 2011; Šíchová et al. 2015; Lukhtanov et al. 2018).

### Filtering and Polarization of SNPs

Allele counts for each population were obtained using *VCFtools* v. 0.1.15 (Danecek et al. 2011). Only nonexonic, biallelic SNPs with no missing data for any individual, and in regions not masked by *RepeatMasker* in the *L. sinapis* reference assembly (Talla et al. 2017; Talla, Johansson, et al. 2019), were kept for downstream analyses. The rationale behind excluding exonic SNPs was to minimize the impact of selection on the allele frequencies, and SNPs in repetitive regions were excluded because of the reduced ability for unique read mapping (Sexton and Han 2019), and their higher potential for ectopic gene conversion, which deserve a separate treatment (Roy et al. 2000; Chen et al. 2007). Sex chromosome-linked SNPs were considered like any other SNP. The lack of recombination in female meiosis in butterflies (Maeda 1939; Turner and Sheppard 1975; Suomalainen et al. 2009) and the reduced effective population size (*N*_e_, three Z chromosomes per four autosomes [*A*]) cancel out (Charlesworth 2012). This leaves only their relative recombination rate (*r*) affecting intensity of gBGC (*B*), assuming that effective sex ratios are equal, and that conversion tract length (*n*) and transmission bias (*c*) are identical for Z and A,

$$\frac{B_{Z}}{B_{A}}=\frac{3N_{e}\;\mathrm{nc}r_{Z}\;\frac{2}{3}}{4N_{e}ncr_{A}\;\frac{1}{2}}=\frac{r_{Z}}{r_{A}}.$$

SNPs were polarized using invariant sites in one or two outgroup populations, again allowing no missing data (supplementary table S1, Supplementary Material online). We denote this polarization scheme “strict.” We also tested a more “liberal” polarization approach where only the individual with the highest average read depth per outgroup population was used to polarize SNPs, allowing for one missing allele per individual. Mean read depth per individual was obtained using *VCFtools* v. 0.1.15 (Danecek et al. 2011). The liberal polarization scheme was mainly used to test the impact of polarization on estimation of the mutation bias (λ) of S→W mutations over W→S mutations (supplementary table S1, Supplementary Material online). The “strict” polarization was used for all analyses unless otherwise stated. We considered alternative (i.e., not in the reference genome) alleles as the ancestral allele if all outgroup individual(s) were homozygous for that allele (“strict” polarization and “liberal” polarization).

Derived allele frequency spectra of segregating variants were computed for the following categories of mutations; GC-conservative/neutral (S→S and W→W, collectively denoted N→N), strong to weak (S→W), and weak to strong (W→S). All alternative alleles inferred as ancestral alleles were used to replace the inferred derived reference allele to make a model of an ancestral genome using *BEDTools* v. 2.27.1 *maskfasta* (Quinlan & Hall 2010). This method leverages the information from invariant sites in all sequenced individuals to decrease the reference bias when calculating GC content. However, the ancestral genome was biased towards *L. sinapis* given that it both served as a reference genome and had more polarizable SNPs than the *L. reali* and *L. juvernica* populations (supplementary table S1, Supplementary Material online).

### Inferring GC-Biased Gene Conversion from the DAF Spectrum

To estimate the strength of gBGC, we utilized a population genetic maximum likelihood model (Muyle et al. 2011; Glémin et al. 2015), implemented as a notebook in *Mathematica* v. 12.0 (Wolfram Research 2019). The model jointly estimates the S→W mutation bias (λ) and the population-scaled coefficient of gBGC (*B *=* *4 *N*_e_*b*), in which *b* is the conversion bias. To account for demography, the model introduces a nuisance parameter (*r_i_*) per derived allele frequency class (*i*), except singletons, following Eyre-Walker et al. (2006). The model also estimates the genetic diversity of N→N and W→S spectra (θ_N_ and θ_WS_, respectively) and computes an estimate of the skewness of S→W and W→S alleles in the folded site frequency spectrum. We applied four of the implemented models, that is, M0, M0*, M1, and M1*, as the more extended models have large variance without prior information on heterogeneity of recombination intensity at a fine scale (Glémin et al. 2015), which is currently lacking for Lepidoptera. The M0 model is a null model that evaluates the likelihood of the observed DAF spectrum for a population genetic model without gBGC (i.e., *B *=* *0). M1 extends this model by including gBGC via the parameter *B*. M0* and M1* are extensions of M0 and M1, respectively, where one additional parameter per mutation class is incorporated, to account for polarization errors. We analyzed separately all nonexonic sites, and excluding- or including ancestral CpG-prone sites, meaning trinucleotides including the following dinucleotides: CG, TG, CA, NG, TN, CN, NA centered on the polarized variant. N here means either a masked or unknown base. Following Glémin et al. (2015), we used GC content as a fixed parameter in the maximum likelihood estimation. GC content in the repeat- and gene-masked ancestral genome model was determined by the *nuc* program in the *BEDTools* v.2.27.1 suite. Coordinates of repeats and exons (including introns and UTR regions if available) were obtained from Talla et al. (2017) and Leal et al. (2018), respectively. The number of G and C bases at ancestral CpG-prone sites was computed using a custom script and subtracted from the GC of all nonexonic sites to obtain the GC content for the set excluding ancestral CpG-prone sites.

### GC Centiles

The polarized nonrepetitive, nonexonic SNPs of each population were divided into 100 ranked bins based on local GC content (GC centiles) in the repeat- and exon-masked ancestral genome. This means, all GC centiles represented unequally sized chunks of the genome with equal numbers of polarizable SNPs. The GC content was estimated in 1 kb windows of the reconstructed and repeat- and exon-masked ancestral genome (described above) using *BEDTools* v. 2.27.1 *nuc* (Quinlan and Hall 2010), correcting for the number of N bases. To calculate the overall GC content of a centile, we summed the GC content of each 1 kb window. Separate DAFs were created per centile and parameters of gBGC and mutation bias were estimated with the models previously described. We also estimated the genetic diversity per GC centile and population using the average pairwise differences (nucleotide diversity, π), and excluded masked bases when averaging. We calculated π for all sites without any missing data, separately for each population, using 1 as value for the max missing (-mm) parameter in the *–site-pi* function of *VCFtools* v. 0.1.15 (Danecek et al. 2011). We also calculated separate π for polarized sites belonging to the following mutation categories (S→S), (W→W), (S→W), and (W→S) for each population and centile, using a custom function in *R* (R Core Team 2020), based on the following parameterization,

$$\pi_{\mathrm{obs}}^{\mathrm{XY}}=\frac{\sum_{i=1}^{n-1}i(n-i)x_{i}^{\mathrm{XY}}}{\left(\begin{array}{c} n\\ 2 \end{array}\right)L_{X}},$$

where *n* is the sample size and *x_i_*^XY^ is the number of sites with the *i*th derived allele frequency for mutations from X→Y with X, Y ∈ {W, S}. $L_{X}$ is the number of AT or GC sites in a certain centile for alleles of W or S origin, respectively.

To average π, we used the number of unmasked bases within the range of GC values defined by each centile. The proportion of coding bases (CDS density) was used as a proxy for the impact of linked selection in general, and background selection in particular. CDS density was estimated separately for each population and centile by aggregating the CDS content across all 1 kb windows for a particular centile. A custom-made script was used to assess the impact of read depth on the pattern of π across GC centiles. This script combined *BEDTools* v. 2.27.1 (Quinlan and Hall 2010) *complement*, *genomecov*, and *intersect* to calculate the read depth per unmasked base pair. Average read-depth per individual and centile was then plotted against GC content to qualitatively assess if the population-specific patterns followed what was observed for the association between π and GC.

### Model of the Effect of gBGC and Mutation Bias on Genetic Diversity

We consider a model in which the effect of gBGC (*B*) and mutation bias (λ) determines the level of π relative to a reference case where *B *=* *0 (McVean and Charlesworth 1999). For this purpose, we first define diversity π as the weighted sum of the following mutation categories (S→W), (W→S), (S→S), and (W→W),

$$\pi =x_{\mathrm{GC}}\pi^{\mathrm{SW}}+(1-x_{\mathrm{GC}})\pi^{\mathrm{WS}}+x_{\mathrm{GC}}\pi^{\mathrm{SS}}+(1-x_{\mathrm{GC}})\pi^{\mathrm{WW}}.$$

Then, under the assumption that GC-conservative/neutral mutations are equal and can be summarized by N→N diversity,

$$\pi =x_{\mathrm{GC}}\pi^{\mathrm{SW}}+(1-x_{\mathrm{GC}})\pi^{\mathrm{WS}}+\pi^{\mathrm{NN}}.$$

Next, we let *x*_GC_ represent the equilibrium GC content determined by gBGC (*B*) and opposing mutation bias (λ) (Li et al. 1987; Bulmer 1991; Muyle et al. 2011),

$$x_{\mathrm{GC}}=\frac{1}{1+\lambda e^{-B}},$$

and introduce relative diversity $\pi_{\mathrm{rel}}$ that is standardized for the reference case (*B *=* *0).

$$\pi_{\mathrm{rel}}=\frac{2\lambda x_{\mathrm{GC}}\left(\frac{1}{1-e^{B}}+\frac{1}{B}\right)+2\left(1-x_{\mathrm{GC}}\right)\left(\frac{1}{1-e^{-B}}-\frac{1}{B}\right)+\frac{\theta_{N}}{\theta_{\mathrm{WS}}}}{\frac{2\lambda }{1+\lambda }+\frac{\theta_{N}}{\theta_{\mathrm{WS}}}}.$$

From an empirical perspective this means that π_rel_ is the predicted π relative to the reference case (*B *=* *0) when the observed GC content is at a value determined by gBGC and mutation bias (1/1* *+* *λe^−^^*B*^). Here, the numerator of the equation for $\pi_{\mathrm{rel}}$consists of three terms each describing the relative contributions of S→W, W→S, and N→N mutations. GC-changing mutations have a diversity determined by λ and *B*, whereas the contribution of GC-conservative/neutral mutations is affected by the ratio of N→N diversity (θ_N_) over W→S diversity (θ_WS_). The model assumes gBGC–mutation–drift (GMD) equilibrium.

Fitting the GMD model to data relies on obtaining a neutral reference π value unaffected by demographic fluctuations in population size, selection, or gBGC. Such a value is unattainable, except for the most well-studied model organisms (Pouyet et al. 2018). Maximum observed genetic diversity, π_max_, could be used as a proxy for neutral diversity which should be reasonable if the entire genome is reduced below the neutral value through linked selection (Torres et al. 2020). Another approach, which we employ here, is to fit the model without estimating a neutral reference π. This allows us to estimate how *B*, λ, and the relative amount of GC-changing mutations affect π_rel_ when GC content equilibrium is reached.

For the GMD model, we can also define π_rel_ values for the separate mutation categories.

$$\pi_{\mathrm{rel}}=x_{\mathrm{GC}}\pi_{\mathrm{rel}}^{\mathrm{SW}}+{(1-x_{\mathrm{GC}})\pi }_{\mathrm{rel}}^{\mathrm{WS}}+{x_{\mathrm{GC}}\pi }_{\mathrm{rel}}^{\mathrm{SS}}+(1-x_{\mathrm{GC}})\pi_{\mathrm{rel}}^{\mathrm{SS}}.$$

This means,

$$\begin{array}{c} \pi_{\text{rel}}^{\text{SW}}=\frac{2\lambda \left(\frac{1}{1-e^{B}}+\frac{1}{B}\right)}{\frac{2\lambda }{1+\lambda }+\frac{\theta_{N}}{\theta_{\text{WS}}}},{\;\pi }_{\text{rel}}^{\text{WS}}=\frac{2\left(\frac{1}{1-e^{-B}}-\frac{1}{B}\right)}{\frac{2\lambda }{1+\lambda }+\frac{\theta_{N}}{\theta_{\text{WS}}}},\\ \pi_{\text{rel}}^{\text{SS}}=\frac{\frac{\theta_{\text{SS}}}{\theta_{\text{WS}}}}{\frac{2\lambda }{1+\lambda }+\frac{{}_{N}}{{}_{\text{WS}}}},{\;\pi }_{\text{rel}}^{\text{WW}}=\frac{\frac{\theta_{\text{WW}}}{\theta_{\text{WS}}}}{\frac{2\gamma }{1+}+\frac{\theta_{N}}{\theta_{\text{WS}}}} \end{array}$$

These equations provide expectations for how the π_rel_ of S→W, W→S, S→S, and W→W mutations vary with GC content.

### Statistical Analyses

All statistical analyses were performed using *R* v. 3.5.0-4.0.2 (R Core Team 2020). Linear models and correlations were performed using default packages in R. We analyzed the relative contribution of GC content and CDS density to variation in π per mutation category using quadratic regressions:

$$\pi_{\mathrm{obs}}^{\mathrm{XX}}\;\sim \;\mathrm{GC}\;\mathrm{content}+{\left[\mathrm{GC}\;\mathrm{content}\right]}^{2}+\mathrm{CDS}\;\mathrm{density}+{\left[\mathrm{CDS}\;\mathrm{density}\right]}^{2}+\mathrm{GC}\;\mathrm{content}:\mathrm{CDS}\;\mathrm{density}.$$

We performed model reduction such that insignificant predictor terms where dropped until only significant terms remained. Phylogenetic independent contrasts (Felsenstein 1985) were performed using the *pic()* function in the package *ape* (Paradis and Schliep 2019). This package was also used to depict the phylogeny in figure 1*A*. Other plots were either made using base *R* or the *ggplot2* package (Wickham 2016).

## Supplementary Material

Supplementary data are available at *Genome Biology and Evolution* online.

## Supplementary Material

evab064_Supplementary_Data
