# Supplementary material for: The Effects of GC-Biased Gene Conversion on Patterns of Genetic Diversity among and across Butterfly Genomes
Source: Genome Biol Evol. 2021 Mar 24;13(5):evab064. doi: 10.1093/gbe/evab064 (PMC8175052; doi:10.1093/gbe/evab064)
Supplement: evab064_Supplementary_Data [file evab064_supplementary_data.zip › gBGC_diversity_figure_legends.docx]

**Figure 1. *Leptidea* butterflies show variation in the genome-wide strength of gBGC**. A) Phylogeny of the six *Leptidea* populations included in this study. Node values represent support from 100 bootstrap replicates on sites. The phylogeny in A) is based on a subtree from a maximum-likelihood phylogeny used as a starting tree in Figure 1 of Talla *et al.* (2017). A mounted specimen of a *Leptidea sinapis* is shown. B) Estimates of the population-scaled coefficient of gBGC (*B =* 4*N_e_b*). Circles represent point estimates from the original DAF spectra using model M1*, bars are mean values of *B* for the 1,000 bootstrap replicates on segregating sites. Overlain and opaque violins are bootstrapped values for model M1* and underlain, transparent violins are estimates for model M1.

**Figure 2 Determinants of variation in the strength of gBGC among populations** A) Relationship between π and *B*. B) Relationship between diploid chromosome number and *B* (M1*). Points in B) show lowest and highest estimate of diploid chromosome number for each population. Colors represent the populations shown in Figure 1. Insets in A) and B) show phylogenetically independent contrasts of each respective axis variable based on the phylogeny in Figure 1A. Contrasts for diploid chromosome number were based on midpoint value.

**Figure 3. Relationship between *B,* λ and observed GC content in the ancestral genome.** A) Association between *B* and observed GC content in the ancestral genome for the *L. sinapis*-*L. reali* clade, and B) for the *L. juvernica* populations. Higher GC content was significantly consistent with greater *B* in all populations except Spa-rea and Ire-juv. C) Relationship between λ and GC content was negative for all populations in the *L. sinapis*-*L. reali* clade. D) Shows the same as C) but for the *L. juvernica* populations. Neither Kaz-juv nor Ire-juv showed significant associations between λ and GC content. Lines in plots represent significant linear regressions performed separately per population between the X- and Y variables.

**Figure 4. Observed GC content, equilibrium GC content and their association with λ, *B* and genetic diversity (π)**. A) Observed GC content compared to equilibrium GC content determined by mutation bias (λ) alone. B) Observed GC content compared to equilibrium GC content when accounting for gBGC. Dotted lines in (A) and (B) represent x = y. C) The skewness of the folded SFS shows the strong S🡪W bias in the segregating variation which increases with observed GC content in the ancestral genome. Extrapolating from the distribution of skewness values onto the y=0 line serves as a validation of the estimated λ. Dotted vertical lines represent the GC equilibrium under mutation bias alone, 1/(1+λ), for each population. D) The association between genetic diversity (*π*) and observed GC content. Points in all panels represent GC centiles.

**Figure 5: A model for genetic diversity under gBGC-mutation-drift equilibrium, predicted π_rel_ per population and π per mutation category.** A) Genetic diversity relative to neutral (*B* = 0) across equilibrium GC content determined by *B* and λ. Lines begin at *B =* 0 and end at *B* = 8. The mutation bias is held constant. B) Genetic diversity values predicted from the gBGC-mutation-drift equilibrium model using output from the inference of gBGC. Most of the genome for each population have values of *B* and λ such that their relative strength boosts the long-term genetic diversity compared to *B* = 0. The lower and upper limit of the box correspond to the first and third quartiles. Upper and lower whiskers extend from the top- and bottom box limits to the largest/smallest value at maximum 1.5 times the inter-quartile range. C) Components of the gBGC mutation drift model. Only results from λ = 3 are shown. The separate mutation categories were standardized by mutational opportunity while “All” was standardized as in A). The genetic diversity is here assumed to be equal for N🡪N and W🡪S mutations (θ_N_ / θ_WS_ = 1). D) Genetic diversity in Swedish *L. sinapis* measured by average pairwise differences (π) across genomic GC content for all four mutation categories: S🡪S (SS), S🡪W (SW), W🡪S (WS), W🡪W (WW). The other populations are shown in Figure S4.

**Figure 6: Relationship between π, CDS density and GC content**. A) shows the relationship between CDS density and GC content for Swe-sin in four nonoverlapping equidistant intervals of GC content. B) instead shows the relationship between π and CDS density in the same bins separately for: S🡪S, W🡪W, S🡪W and W🡪S mutations. The fifth GC content bin is not shown because it includes only one centile. See Figure S5 for the other populations. *R^2^ =* proportion of variation explained, *k* = slope of regression (times 10^3^ for readability in B). GC bins 1-4 shown left to right. Mutation categories from top to bottom row: S🡪S, W🡪W, S🡪W and W🡪S.
